# Transmyringeal ventilation tube insertion for unilateral Menière’s disease: a protocol for a prospective, sham-controlled, double-blinded, randomized, clinical trial

**DOI:** 10.1186/s13063-022-06777-w

**Published:** 2022-10-17

**Authors:** Casper Grønlund Larsen, Mikael Karlberg, Frank Guldfred, Louise Devantier, Mathias Maagaard, Preben Homøe, Bjarki Ditlev Djurhuus

**Affiliations:** 1 Department of Otorhinolaryngology and Maxillofacial Surgery, Zealand University Hospital, Køge, Denmark; 2 Department of Otorhinolaryngology, Head and Neck Surgery, Skåne University Hospital, Lund, Sweden; 3 Ear, Nose, and Throat Private Practice, Roennede, Denmark; 4 Department of Clinical Medicine, Aarhus University, Aarhus, Denmark; 5 Department of Anaesthesiology, Zealand University Hospital, Køge, Denmark

**Keywords:** Randomized controlled trial, Ventilation tube insertion, Menière’s disease, Menière

## Abstract

**Background:**

Menière’s disease is an idiopathic disorder characterized by recurrent episodes of vertigo lasting more than 20 min, unilateral sensorineural hearing loss, and tinnitus. If vertigo attacks occur frequently, the patient is usually severely incapacitated. Currently, there is no consensus on the treatment of Menière’s disease. The evidence regarding most treatment options is sparse due to a lack of randomized trials together with an often-spontaneous relief over time and a considerable placebo effect. Insertion of a transmyringeal tube is a simple and relatively safe, minimally invasive procedure and previous open-label trials have shown promising results.

**Study design:**

This is a prospective, sham-controlled, double-blinded, randomized, clinical trial.

**Aim:**

This trial aims to assess the effects of inserting a ventilation tube into the tympanic membrane compared with sham treatment for definite or probable unilateral Menière’s disease according to the criteria formulated by the Classification Committee of the Bàràny Society.

**Outcomes:**

The primary outcome will be the number of spontaneous vertigo attacks lasting more than 20 min and time to treatment failure. In addition to the primary outcome, we will assess various secondary outcomes related to hearing, ear fullness, dizziness, and serious adverse events.

**Sample size:**

An estimated 104 participants in total or 52 participants in each group will be necessary.

The primary analysis will be according to the intention-to-treat principle. The trial will be initiated in 2021 and is expected to end in 2025.

**Trial status:**

ClinicalTrials.gov: NCT04835688. Registered on April 8, 2021.

Protocol version: 1.8, 26-09-2022. Date of first enrollment: October 1st, 2021. End of study: anticipated January 2025.

**Supplementary Information:**

The online version contains supplementary material available at 10.1186/s13063-022-06777-w.

## Introduction

### Background information and rationale

Menière’s disease is an inner ear disorder with recurrent attacks of vertigo, fluctuating sensorineural hearing loss, tinnitus, and aural fullness [1]. The underlying pathogenetic mechanisms are not known. The pathologic-anatomic correlate of the disease is endolymphatic hydrops, i.e. distension of the endolymphatic spaces as seen at post-mortem microscopic examination of the temporal bone. Prevalence figures are in the range between 0.1 and 0.5% in the population [2, 3]. In Denmark, the estimated prevalence of Menière’s disease is 3500 [4]. The disease commonly begins in the fourth or fifth decade of life, and the prevalence increases with age [5].

There are a great number of different treatment options for Menière’s disease including diuretics, sodium restriction, beta-histidine, and psycho-supportive means, most of which are not validated [6]. The only validated treatment for vertigo attacks is chemical labyrinthectomy by intra-tympanic injections of the ototoxic antibiotic gentamicin for which two double-blind, placebo-controlled trials found a significant effect [7, 8]. Treatment with gentamicin is ablative, i.e. the goal of the treatment is to destroy the vestibular sensors of the affected ear. This carries a risk of long-standing unsteadiness alongside a permanent hearing loss in the treated ear. Still, no treatments seem to protect from the hearing loss associated with Menière’s disease.

The first to advocate the use of transmyringeal ventilation tubes for Menière’s disease was Tumarkin in 1966 [9]. Tumarkin et al. suggested that negative middle-ear pressure, due to poor tubal function, would lead to a relative over-pressure in the inner ear and that this might be one of the mechanisms behind Menière’s disease. Also, Tumarkin et al. presented several cases where treatment with transmyringeal tubes resulted in relief from vertigo attacks. Hall and Brackmann performed tympanometry in patients with Menière’s disease and showed that some, but not all, patients had negative middle-ear pressure and they questioned Tumarkin’s suggestions [10]. Recent results from Brattmo et al. on long-term measurement of middle-ear pressure and tubal function showed that a majority of patients with Menière’s disease have a poor tubal function [11].

#### Choice of comparator

Open-label trials have shown promising results with about 80–90% of the patients showing significant relief from vertiginous spells during treatment with transmyringeal tubes. In one randomized, single-blinded, controlled trial the long-term effect over 12 months of transmyringeal tubes was compared to that of endolymphatic sac surgery, and no significant differences between the treatments were found [12]. Animal studies have shown that the insertion of a transmyringeal tube prevents the induction of endolymphatic hydrops after ligation of the endolymphatic duct [13]. However, the placebo effect associated with MD treatment is generally considered to be substantial. Therefore, we find it relevant to do a sham-controlled trial. When planning a randomized clinical, placebo-controlled trial, AAO-HNS recommends having a follow-up time of 2 years or more [14].

Every year, a lot of children in Denmark and Sweden are treated with transmyringeal tubes to prevent acute purulent otitis media and to relieve conductive hearing loss due to secretory otitis media. An unknown number of tubes are inserted in adults because of secretory otitis media, tuba aperta, and to treat Menière’s disease. Tube insertion in adults is usually easily performed as an office procedure under topical or local anaesthesia. The main complications from ventilation tubes are purulent otitis media while the tube is in place and persistent perforation after tube extrusion.

### Objectives

#### Main objective

The main objective of the trial is to assess the effects of transmyringeal ventilation tubes compared with sham treatment which does not ventilate the middle ear.

#### Research hypothesis

Insertion of a ventilation tube does have an impact on vertigo attacks in patients with Menière’s disease.

### Trial design

The design is a parallel, prospective, sham-controlled, double-blinded, randomized, clinical trial.

We plan to test superiority using the intention-to-treat set. We propose declaring surgical management superior to sham treatment, only if shown to be superior using the intention-to-treat analysis set.

## Methods: participants, interventions, and outcomes

We used the SPIRIT reporting guidelines when developing the protocol [15]. A flow diagram for the study can be found in the Additional file 1: Appendix.

### Study setting

The study will be conducted at an estimated 40 private-practising ear, nose, and throat-clinics (ENT-clinics) in Denmark and Skåne University hospital in Sweden.

### Eligibility criteria

#### Study subjects

A minimum of 94 participants with definite or probable unilateral Meniére’s disease will be enrolled according to the criteria formulated by the Classification Committee of the Bàràny Society. But to get safety margins and account for drop-outs, we have decided to enrol a total of 104 consecutive participants.

#### Inclusion criteria

Participants aged 18 years or older with definite or probable unilateral Menière’s disease according to the diagnostic criteria formulated by the Classification Committee of the Bárány Society, The Japan Society for Equilibrium Research, the European Academy of Otology and Neurotology (EAONO), the Equilibrium Committee of the American Academy of Otolaryngology – Head, and Neck Surgery (AAO-HNS), and the Korean Balance Society [16]:

A. Two or more spontaneous episodes of vertigo, each lasting 20 min to 12 h
B. Audiometrically documented low- to medium-frequency sensorineural hearing loss in the affected ear on at least one occasion before, during, or after one of the episodes of vertigo
C. Fluctuating aural symptoms (hearing, tinnitus, or fullness) in the affected ear
D. Not better accounted for by another vestibular diagnosis

Furthermore, the patient must have experienced at least two vertigo attacks during the last 3 months before inclusion.

In probable Menière’s disease, the diagnostic criteria cover the same points (A, C, D). However, episodes of vertigo or dizziness may last from 20 min to 24 h [17]. Furthermore, the patients must have experienced at least two spontaneous vertigo attacks lasting more than 20 min during the past 3 months [16].

#### Exclusion criteria

- Bilateral Menière’s disease
- Previous treatment with transmyringeal ventilation tubes after childhood
- Previous ablative or surgical therapy, such as intratympanic gentamicin or endolymphatic sac surgery
- Expected problems to adhere to the study protocol (dementia, non-fluent in Danish, substance abuse, etc.)

### Interventions

#### Description

The participants will be divided randomly into two intervention arms, an experimental group, and a control group. The procedures are described below.

In both groups, the tympanic membrane will be anaesthetized by local application of topical prilocaine (EMLA) or phenol or by infiltration anaesthesia of the outer ear canal. The choice of method is left to the ear, nose, and throat specialist (ENT specialist).

For the experimental group, insertion of a ventilation tube will be performed. An incision is performed, usually in the lower, anterior quadrant of the tympanic membrane, and the transmyringeal tube is inserted. This procedure is usually painless and well-tolerated.

For the control group, the ENT specialist will touch the tympanic membrane with an alligator ear forceps to simulate getting a paracentesis. In the same procedure, a ventilation tube is placed on the tympanic membrane and removed again afterwards without having made a paracentesis. The reason for the above-mentioned is to simulate getting a paracentesis and insertion of a ventilation tube.

The interventions will be performed after the participant has been included in the trial. To improve adherence to the intervention, the position of the tube will be inspected once every month for the first 3 months. This is to avoid spontaneous extrusion of the tube.

If the tube is spontaneously extruded, a new ventilation tube insertion will be performed. In the same way, if a patient with a sham intervention thinks that the tube has spontaneously extruded, then a new sham procedure will be performed.

#### Modifications

We do not offer a cross-over treatment in this study as the episodes of vertigo are fluctuating in nature with a potential difference in “baseline” characteristics before and after cross-over treatment which makes it difficult to compare. Furthermore, it is not possible to modify a ventilation tube insertion if the participant is unsatisfied with the treatment, in response to harms, or as a participant request. Therefore, if the participant is unsatisfied with the current treatment, then he/she has the right to drop out of the study.

#### Adherence

The participant will be followed once every month for the first 3 months to improve the adherence to the intervention and to perform tympanometry to detect if spontaneous extrusion of the ventilation tube has occurred.

#### Concomitant care

Any medication for Meniere disease that a patient takes at inclusion is continued with unchanged doses during the study period. Medications, such as anti-histamine suppositories, to alleviate acute vertigo attacks are permitted. The use of such rescue medications is recorded by the patient.

Other relevant treatment options for patients suffering from Menière’s disease (i.e. Meniett-treatment, intratympanic steroid injection, or surgery) are not allowed during the trial.

### Outcomes

#### Primary outcome (main study 3 months)

The number of spontaneous vertigo attacks lasting more than 20 min during the study period will be compared between the two study groups.

This outcome was chosen as vertigo is the most disabling symptom of Menière’s disease.

#### Secondary outcomes (main study 3 months)

The secondary outcomes of this study are comparisons between the two study groups regarding:

- Pure-tone audiometry of affected ear, four-tone average of 500, 1000, 2000, and 3000 Hz (dB)
- Pure-tone audiometry of affected ear, three-tone average of 125, 250, and 500 Hz (dB)
- Speech audiometry of affected ear, discrimination (%)
- AAO-HNS Functional level scale (1–6)
- Subjective hearing (0–10 arbitrary scale)
- The subjective intensity of ear fullness/pressure (0–10 arbitrary scale)
- The subjective intensity of dizziness/unsteadiness (0–10 arbitrary scale)
- The subjective intensity of tinnitus (0–10 arbitrary scale)
- Need of “escape medication”
- Number of subjects leaving the study because of treatment failure
- Number of subjects satisfied with the treatment
- Serious adverse events (as defined by the ICH-GCP)

These outcomes were chosen to assess any possible effects of treatment on hearing, functional level, and subjective symptoms.

The number of vertigo attacks, need for escape medication, and level of subjective symptoms will be recorded weekly by the study subject on a standardized form. Hearing tests will be performed at inclusion and after 3 months. A tympanometry will be performed after 1, 2, and 3 months. Assessment of AAO-HNS functional level scale will be made at inclusion and after 3 months. Menière’s disease staging (I–IV) will be decided at inclusion. Four-tone average of worst audiogram during the last 6 months: < 26 dB = Stage 1, 26–40 dB = Stage 2, 41–70 dB = Stage 3, > 70 dB = Stage 4. The equipment for hearing tests is maintained and calibrated by each study centre.

#### Primary outcome (the extended study of up to 24 months)

Time to treatment-failure will be compared between the two study groups and presented as a Kaplan-Meier plot.

### Participant timeline

The participants will be followed for 3 months after the start of treatment with follow-up visits at (Table 1):

- One month ± 4 days
- Two months ± 4 days
- Three months ± 4 days

**Table 1:** Participant timeline

	Study period							
	Enrolment	Allocation	Post-allocation					Close-out
Timepoint	-t_1_	0	t_1_	t_2_	t_3_	t_4_	t_5_	t_x_
**Enrolment**								
Eligibility screen	X							
Informed consent	X							
Allocation		X						
**Interventions**								
Ventilation tube insertion		X						
Sham-treatment		X						
**Assessments**								
Subjective symptom score-scheme^a^		X	X	X	X			
AAO-HNS functional level scale		X			X	X	X	
Pure-tone and speech audiometry		X			X			

**Timeline:**

t_1_: At one month

t_2_: At two months

t_3_: At three months

t_4_: At three to six months from t_3_

t_5_: At three to six months from t_4_

t_6_: At three to six months from t_5_

t_7_: At three to six months from t_6_

t_8_: At three to six months from t_7_

t_9_: At three to six months from t_8_

^a^Patients will be asked to fulfill a weekly subjective symptom score-scheme regarding dizziness, tinnitus, hearing and aural fullness.

The participants will then be asked if they want to participate in a follow-up study which entails a follow-up every 3 to 6 months for a maximum of 24 months.

**Table:** 

4 mandatory visits:	Inclusion, month 1, month 2, month 3			
**Visit**	**Inclusion**	**Month 1**	**Month 2**	**Month 3**
Inclusion assessment	**X**			
Informed consent	**X**			
AAO-HNS Function Level Scale	**X**			**X**
Tympanometry	**X**	**X**	**X**	**X**
Pure tone audiometry	**X**			**X**
Speech audiometry	**X**			**X**
Weekly subj. symptom form	X	**X**	**X**	**X**
Placement of tube or sham	**X**			

### Sample size

The primary outcome in the study will be the number of attacks within the first 3 months after ventilation tube insertion. Because this is count data (i.e. an incidence rate) a Poisson distribution is expected, and a Poisson regression model most likely will give the best fit for analysis of the primary outcome.

The simulation software ”Powersim” developed for the used statistical package, STATA, was applied for Power calculations.

Given the lack of published data concerning the expected effect of ventilation tube insertion, we chose to assume a 50% reduction in the intervention group, which we believe is realistic as well as a necessity. A 50% reduction gives an Incidence Rate Ratio (IRR) of 0.5 and an ln(IRR) of −0.69315.

Using an alpha value of 0.05 and an expected effect size of 0.5 (ln(0.5) = −0.69315), and to obtain a power of 0.8, an expected 94 participants in total or 47 participants in each group will be necessary. To adjust for drop-outs, a safety margin of 10% results in 104 participants in total.

### Recruitment

We have been in contact with DØNHOF (the danish ear, nose, throat-specialists organization), and DØNHOF is willingly conveying this contact by sending e-mails as well as bringing information about the project on their web page (http://www.doenho.dk). Besides, we will present the project at relevant conferences such as DSOHH (the Danish Organization of Ear, Nose, Throat, and Head Neck Surgery) as well as DSFV (the Danish Organization of Vestibulogy). At this point, we have received permission from the Committee on Health Research Ethics to perform the trial. Therefore, we are about to initiate contact with the ENT specialists. Hence, we do not know the exact locations and responsible ENT specialists yet. As soon as it is clarified which clinics will be participating in the study, it will be announced by an additional protocol for the Committee on Health Research Ethics.

Participants will be enrolled in cooperation with private-practising ENT specialists in Denmark and Sweden. The ENT specialists will be given a scheme on inclusion criteria to include relevant participants. An estimated 104 participants are needed in cooperation with an estimated 40 ENT specialists. We expect each ENT clinic to enrol 2.5 participants per year on average.

## Methods: assignment of interventions

### Allocation

#### Sequence generation

Once informed consent has been obtained, the participant will be randomly assigned to either a sham or experimental group with a 1:1 allocation without site-stratification as per a computer-generated randomization schedule. An independent statistician will generate the allocation sequence.

#### Concealment mechanism

Participants will be randomized using REDCap, a web-based randomization service. The allocation concealment will be ensured, as the service will not release the randomization code until the patient has been recruited into the trial, which takes place after all baseline measurements have been completed in the RedCap system.

#### Implementation

The statistician will generate the allocation sequence. The ENT specialists will screen and obtain informed consent. The ENT specialist will be given a direct phone number to a project nurse who will perform the registration and randomization of the participant.

### Blinding

#### Masking

Participants, care providers, data collectors, outcome assessors, research personnel, and data analysts will be blinded to treatment allocation. However, the ENT specialist performing the insertion of the ventilation tube or sham treatment will inevitably be aware of treatment allocation.

At the end of the final visit, the participants will be asked what treatment they believe to have received. This will enable an assessment of the adequacy of blinding.

#### Emergency unblinding

The treatment allocation of a participant may be unblinded in case of an emergency where the treating physician is unable to adequately treat the participant without being aware of treatment allocation. The investigator will be notified, and the investigator will contact the data-management team for unblinding. The reason for unblinding will be documented.

### Data collection plan

Pure-tone audiometry, speech audiometry, and assessment of AAO-HNS (the Committee on Hearing and Equilibrium of the American Academy of Otolaryngology – Head and Neck Surgery) functional level are performed when a participant enters the study and is repeated after 3 months of treatment. Following inclusion, a weekly questionnaire on symptoms will be filled out by the participants.

#### Audiometric tests

The audiometric tests include pure-tone audiometry: an average of 500, 1000, 2000, and 3000 Hz and an average of 125, 250, and 500 Hz (dB), speech audiometry: single-syllable phonetically balanced word lists, speech discrimination (%), and tympanometry (to indicate function and patency of active tubes). The audiometric tests will be performed at inclusion and after 3 months. A tympanometry will be performed after 1, 2, and 3 months to objectively assess tube patency.

#### Functional level scale

The AAO-HNS functional level scale (see Additional file 1: Appendix 5.1) consists of written descriptions of how Menière’s disease affects the life of the participants, from no impact at all (level 1) to handicapped and unable to work (level 6). The participant chooses the description that fits best. The form will be filled out at inclusion and after 3, 6, 12, 18, and 24 months.

#### Self-evaluation of symptoms

Once every week the study subject fills out a form for self-evaluation of symptoms (see Additional file 1: Appendix 5.2). The form includes the number and duration of vertigo attacks longer than 20 min, need and type and dose of escape medication for vertigo, the subjective intensity of dizziness/unsteadiness (VAS scale), subjective hearing loss (VAS scale), the subjective intensity of ear fullness/pressure (VAS scale), and subjective intensity of tinnitus (VAS scale).

All procedures are clinical routine procedures carried out every day at every ENT clinic in Denmark and Sweden, except the assessment of the functional level and filling out forms for self-evaluation of symptoms.

#### Retention

We will try to promote participant retention by following the participant once every month for the first 3 months, then every third month until the end of the trial. We will send e-mails to participants before data collection to remind them of the upcoming data collection. If a participant is discontinued from the trial or withdraws consent and the dropout is before the end of the first 3 months, then the patient will be asked to fill out a functional level scale. If the dropout is after the first 3 months, we will ask the participant if we may continue to record data relevant to the trial.

### Data management

CRF data will be entered into the data management system REDCap. REDCap holds standards according to the Danish Data Protection Agency (i.e. stored on private servers). In the CRFs and the database, participant identification will be replaced by a code, and a participant identification list will be kept safely, separate from the CRFs by the local investigator.

### Statistics

The intervention group and the control group will be compared concerning all outcomes based on the intention-to-treat principle. That is, all participants will be analysed in the groups to which they were randomized, regardless of whether they adhered to the allocated intervention.

In general, count data will be analysed using Poisson regression, continuous data with mixed linear modelling, and binary data with logistic regression. For the speech audiometry, we plan to dichotomize the data into an ≥8% decrease or not, to be able to apply logistic regression. For the AAO-HNS Functional Level Scale, we plan to dichotomize the outcome into a change ≥2 or not, for the same purpose.

#### Outcomes

**Table:** 

**Primary outcome**	**Type of data**	**Analysis**
Number of spontaneous vertigo attacks lasting more than 20 min	Count	Poisson-regression

**Table:** 

**Secondary outcome**	**Type of data**	**Analysis**
Pure-tone audiometry of affected ear, 4 tone average of 500, 1000, 2000, and 3000 Hz	Continuous	Mixed linear modelling
Pure-tone audiometry of affected ear, 4 tone average of 125, 250, and 500 Hz	Continuous	Mixed linear modelling
The subjective hearing	Continuous	Mixed linear modelling
The subjective intensity of ear fullness/pressure	Continuous	Mixed linear modelling
The subjective intensity of dizziness/unsteadiness	Continuous	Mixed linear modelling
The subjective intensity of tinnitus	Continuous	Mixed linear modelling
Number of subjects leaving	Count	Poisson-regression
Number of subjects satisfied	Count	Poisson-regression
The need for escape medication	Count	Poisson-regression
Serious adverse effects	Binary	Logistic regression
Speech audiometry	Binary data. Dichotomized to > 8% or < 8% decrease in speech audiometry	Logistic regression
AAO-HNS Functional Level Scale	Binary. Dichotomized to a change in > 2 or < 2 levels after 3-months follow-up	Logistic regression, alternatively linear regression with bootstrap

#### Additional analysis

We plan to perform a subgroup analysis on the patients who have had a new ventilation tube inserted after spontaneous extrusion of the first ventilation tube.

#### Analysis population and missing data

We plan to test superiority using the intention-to-treat set, considering all patients as randomized regardless of whether they received the randomized treatment. We propose declaring surgical management superior to sham treatment, only if shown to be superior using the intention to treat analysis set. Further analysis can be done using per-protocol analysis.

## Methods: monitoring

### Data monitoring: formal committee

The study is registered at the local branch of the Danish Data Protection Agency in Region Zealand [18] under the ID “REG-035-2021”. The local branch of the Danish Data Protection Agency is independent of the sponsor-investigator and there are no competing interests.

### Data monitoring: interim analysis

An interim analysis is performed on the primary endpoint when 50% of patients have been randomized and have completed the 3 months of follow-up. The interim analysis is performed by an independent statistician, blinded for treatment allocation.

### Harms

#### Adverse events

An adverse event is any untoward medical occurrence reported by the participant, where no relationship between the adverse event and the device under investigation has been judged by the investigator. An adverse device effect is any untoward and unintended response to a medical device. This includes any event resulting from insufficiencies or inadequacies in the instructions for use or the development of the device. This definition also includes any event that is a result of an error. A serious adverse event is an adverse event that led to:

- A death
- A serious deterioration in the health of a subject that resulted in
  - A life-threatening illness or injury
  - Permanent impairment of a body structure or a body function
  - Medical or surgical intervention to prevent permanent impairment to body structure or a body function
  - Required in-patient hospitalization or prolongation of existing hospitalization
- Foetal distress, foetal death or congenital abnormality, or birth defect

A serious adverse device effect is an adverse device effect that has resulted in any of the consequences characteristic of a serious adverse event or that might have led to any of these consequences if suitable actions had not been taken or intervention had not been made or if circumstances had been less opportune.

Foreseeable adverse device effects are mainly purulent otitis media and persistent perforation after tube extrusion. Adverse events and adverse device effects should be assessed from the day of insertion of the tube until week 12 in the main study and until 3 months after end-points (early termination or 24 months) in the follow-up study.

The following events will be reported:

- Purulent otitis media
- Persistent perforation > 3 months after tube extrusion

The following events will not be reported:

- Hospitalization for a procedure that was planned before study participation

### Auditing

Data will be accessible for auditing for the competent authorities such as the Danish Data Protection Agency and the local Committee on Health Research Ethics upon request.

The trial steering group will meet to review trial conduct once every second month. The same process is for the Project Management Group. This process will not be independent from the investigators.

## Ethics and dissemination

### Research ethics approval

We have received approval from the local Committee on Health Research Ethics in Region Zealand with the following ID: SJ-909.

### Ethics statement

We believe that the potential side-effects of this trial are minimal. Sham surgery will always raise ethical considerations. However, ventilation tube insertion is a swift procedure that is performed in local anaesthesia and can be done within a few minutes. The most frequent complications are infection or persistent perforation in the tympanic membrane. This is unlikely to happen in a healthy membrane and can be fixed with eardrops or a myringoplasty. More importantly, treating thousands of patients with ventilation tubes without any beneficial effects are of even greater ethical concern.

It is essential to provide scientific evidence of whether ventilation tubes work in patients with Menière’s disease or not. A negative finding may save patients from ineffective procedures. A positive finding will help patients with Menière’s disease to better disease control, especially in countries where the use of ventilation tube insertion for Menière’s disease is less prevalent. Furthermore, a positive finding may save patients from ablative treatment or invasive surgery such as endolymphatic sac surgery.

### Protocol amendments

We will inform relevant parties if important protocol modifications are made.

### Consent

Information will be provided to the participants by the ENT specialists and not the principal investigator. As it is the ENT specialist who will provide the information, a written contract between the ENT specialist and the principal investigator will be filled out. This contract will contain the following: the name of the ENT specialist who gives the information and receives the informed consent, and a signature from the ENT specialist which confirms that written as well as oral information has been given.

After ensuring that inclusion and exclusion criteria have been met, informed consent will be discussed. Potential participants will be informed both by written as well as oral information about the aim of the investigation, according to the recommendations from the health research ethics committee of Denmark (Videnskabsetisk Komite, Danmark). The participants will receive a leaflet about “their rights as a test person in a biomedical research project” from the health research ethics committee.

When the participant is introduced to the trial for the first time, he or she will be informed by the responsible ENT specialist and be given the written information. The ENT specialist will provide a quiet, undisturbed, and safe place to give the information. Besides, the participant will be offered an information meeting, where the participant may bring an assessor. The information leaflet itself contains details about the trial and its pros and cons and needs to be easy to read for the participant. Furthermore, the participant will be given a link to a webpage about the project (http://www.meniere.dk), where all necessary information will be written as well as a direct e-mail to investigator CGL. The participant will be informed that this is a request for their participation in the study.

The participant will be informed about their right to refuse information about significant health conditions. The physician who gives the information has the responsibility of making sure that the information is understood by the participant. Afterwards, the participant has the right of 24 hours before deciding to participate or not.

On the day of the randomization, the informed consent from the participant will be collected in the paper. The participant will be informed that he or she at any time has the right to withdraw from the trial without affecting current or future treatment and control.

When the results of the trial are available, the sponsor-investigator will inform the patients about the results. Information about participants is protected by the General Data Protection Regulation as well as the Data Protection Act.

### The patient compensation association

The participants in this study will be covered by the danish patient compensation association according to the information given on the website of the Danish National Ethics committee [19].

### Confidentiality

The trial will be conducted according to the regulations of the Danish Data Protection Agency. Only people related to the trial and the central randomization centre will have access to data. Anonymized participant-level data can be requested by researchers.

### Declaration of interests

None.

### Data access

The dataset will be available in a depersonalized format after the end of the trial on the Danish Data Archive. Only the investigator, the sponsor, and the statistician will have access to the final trial dataset.

### Dissemination policy

The Danish Patient Association of Menière’s disease will be informed about the trial as well as the final results. We plan to present our final results at conferences for the Danish Patient Association of Menière’s disease. Trial results will be published in both Danish and English.

The results will be published in a peer-reviewed journal as well as at clinicaltrials.gov and presented at relevant conferences. Both positive, negative, and inconclusive results will be published.

All authorship will be determined according to the International Committee of Medical Journal Editors guidelines for Authorship [20]. The first author is coordinating investigator Casper Grønlund Larsen and the last author is responsible investigator Bjarki Ditlev Djurhuus.

## Supplementary Information

**Additional file 1.**

## References

[1] Espinosa-Sanchez JM, Lopez-Escamez JA (2016). Meniere's disease. Handb Clin Neurol.

[2] Havia M, Kentala E, Pyykko I (2005). Prevalence of Meniere's disease in general population of southern Finland. Otolaryngol Head Neck Surg.

[3] Radtke A, von Brevern M, Feldmann M, Lezius F, Ziese T, Lempert T (2008). Screening for Meniere's disease in the general population - the needle in the haystack. Acta Otolaryngol.

[4] Menière's disease The medical handbook 2018 [Available from: https://www.sundhed.dk/sundhedsfaglig/laegehaandbogen/oere-naese-hals/tilstande-og-sygdomme/indre-oere/menieres-sygdom/.

[5] Stahle J, Friberg U, Svedberg A (1991). Long-term progression of Meniere's disease. Acta Otolaryngol Suppl.

[6] Coelho DH, Lalwani AK (2008). Medical management of Meniere's disease. Laryngoscope.

[7] Stokroos R, Kingma H (2004). Selective vestibular ablation by intratympanic gentamicin in patients with unilateral active Meniere's disease: a prospective, double-blind, placebo-controlled, randomized clinical trial. Acta Otolaryngol.

[8] Postema RJ, Kingma CM, Wit HP, Albers FW, Van Der Laan BF (2008). Intratympanic gentamicin therapy for control of vertigo in unilateral Menire's disease: a prospective, double-blind, randomized, placebo-controlled trial. Acta Otolaryngol.

[9] Tumarkin A (1966). Thoughts on the treatment of labyrinthopathy. J Laryngol Otol.

[10] Hall MC, Brackmann DE (1977). Eustachian tube blockage and Meniere's disease. Arch Otolaryngol.

[11] Brattmo M, Tideholm B, Carlborg B (2012). Inadequate opening capacity of the eustachian tube in Meniere's disease. Acta Otolaryngol.

[12] Thomsen J, Bonding P, Becker B, Stage J, Tos M (1998). The non-specific effect of endolymphatic sac surgery in treatment of Meniere's disease: a prospective, randomized controlled study comparing "classic" endolymphatic sac surgery with the insertion of a ventilating tube in the tympanic membrane. Acta Otolaryngol.

[13] Kimura RS, Hutta J (1997). Inhibition of experimentally induced endolymphatic hydrops by middle ear ventilation. Eur Arch Otorhinolaryngol.

[14] Committee on hearing and equilibrium guidelines for the diagnosis and evaluation of therapy in Meniere's disease. Otolaryngol Head Neck Surg. 1995;113(3):181–5. 10.1016/S0194-5998(95)70102-8. 10.1016/S0194-5998(95)70102-8 7675476

[15] Chan AW, Tetzlaff JM, Gøtzsche PC, Altman DG, Mann H, Berlin JA (2013). SPIRIT 2013 explanation and elaboration: guidance for protocols of clinical trials. BMJ..

[16] Lopez-Escamez JA, Carey J, Chung WH, Goebel JA, Magnusson M, Mandala M (2015). Diagnostic criteria for Meniere's disease. J Vestib Res.

[17] Lopez-Escamez JA, Carey J, Chung WH, Goebel JA, Magnusson M, Mandala M (2017). Diagnostic criteria for Meniere's disease according to the classification Committee of the Barany Society. Hno..

[18] The local branch of the data agency in Region Zealand Region Zealand University Hospitals webpage.: Region Zealand University Hospital; 2021 [A description of the local branch of the data agency in Region Zealand University Hospital.]. Available from: https://www.regionsjaelland.dk/Sundhed/forskning/forfagfolk/faq/Sider/Datatilsyn.aspx.

[19] The danish national ethics committee The danish national ethics committee [Information on the danish compensation association]. Available from: https://www.nvk.dk/emner/forsikring-og-erstatning/vejledning-om-forsikring#1._Generelt_om_forsikring_af_forsøgspersoner.

[20] Defining the Role of Authors and Contributors: ICMJE; [Recommendations]. Available from: https://www.icmje.org/recommendations/browse/roles-and-responsibilities/defining-the-role-of-authors-and-contributors.html.

