# Supplementary material for: Transmyringeal ventilation tube insertion for unilateral Menière’s disease: a protocol for a prospective, sham-controlled, double-blinded, randomized, clinical trial
Source: Trials. 2022 Oct 17;23:877. doi: 10.1186/s13063-022-06777-w (PMC9578195; doi:10.1186/s13063-022-06777-w)
Supplement: Supplementary file 1 — Additional file 1. [file 13063_2022_6777_MOESM1_ESM.docx]

# Appendices

## AAO-HNS functional level scale (14) translated into Danish:

Vælg en af nedenstående muligheder.

| AAO-HNS Ménière’s Disease Functional Level Scale in Danish | |
| --- | --- |
| Sæt ring om tallet, som bedst beskriver, hvordan din dagligdag påvirkes af din svimmelhed | |
| 1 | Min svimmelhed påvirker ikke mine daglige aktiviteter. |
| 2 | Når jeg er svimmel, er jeg nødsaget til at stoppe med hvad jeg er i gang med, men svimmelheden går snart over igen, så jeg kan fortsætte mine aktiviteter. Jeg kan fortsat arbejde, køre bil/cykle og engagere mig i enhver aktivitet, som jeg vælger, uden begrænsninger. |
| 3 | Når jeg er svimmel, er jeg nødsaget til at stoppe med hvad jeg er i gang med, men svimmelheden går over igen, så jeg kan fortsætte mine aktiviteter. Jeg kan fortsat arbejde, køre bil/cykle og engagere mig i de fleste aktiviter, som jeg vælger, men jeg har været nødt til at ændre nogle af mine planer grundet min svimmelhed. |
| 4 | Jeg er i stand til at arbejde, køre bil/cykle, rejse, tage mig af min familie eller engagere mig i de mest vigtige aktiviteter, men jeg må bruge mange kræfter på det. Jeg må konstant foretage ændringer i mine aktiviteter og økonomisere min energi. Jeg kan næsten ikke klare det. |
| 5 | Jeg kan ikke arbejde, køre bil/cykle eller tage mig af min familie. Jeg er ikke i stand til at udføre de fleste af de ting, som jeg plejede at gøre. Selv ved essentielle aktiviteter er jeg begrænset. Jeg er handikappet. |
| 6 | Jeg har været handikappet i et år eller mere og/eller jeg modtager kompensation (økonomisk) grundet min svimmelhed eller balanceproblemer. |
| Monsell EM, et al.: Committee on Hearing and Equilibrium of the American Academy of Otolaryngology – Head and Neck Surgery: Guidelines for the Diagnosis and Evaluation of Therapy in Ménière’s Disease. OtolaryngolHeadNeckSurg1995;113(3):181-185. | |

## Form for weekly symptom assessment in Danish:

**Opfølgningsformular for Menières sygdom**

Patientens nummer i studiet:
Uge (nummer): ……… År: ………

Denne uge har jeg haft …… svimmelhedsanfald som varede længere end 20 minutter.
Hvor længe har anfaldene varet? ……… ……… ………. ……… ……… ……… ……… ……… ………. ………

Jeg har haft behov for at tage medicin mod svimmelhed:
Nej ……… Ja ……… Hvis ja, hvad? ………… Hvor stor dosis? ………

Indsæt et hak på hver af nedenstående linjer, som bedst beskriver, hvor besværet du har
været af svimmelhed, hørenedsættelse, fyldningsfornemmelse og tinnitus den sidste uge:
 **Svimmelhed:**

Ingen 0 1 2 3 4 5 6 7 8 9 10 Værst tænkelige

**Hørenedsættelse:**

Ingen 0 1 2 3 4 5 6 7 8 9 10 Værst tænkelige

**Fyldningsfornemmelse eller trykpåvirkning i øret:**

Ingen 0 1 2 3 4 5 6 7 8 9 10 Værst tænkelige

**Øresusen (tinnitus):**

Ingen 0 1 2 3 4 5 6 7 8 9 10 Værst tænkelige

## Flowchart

##
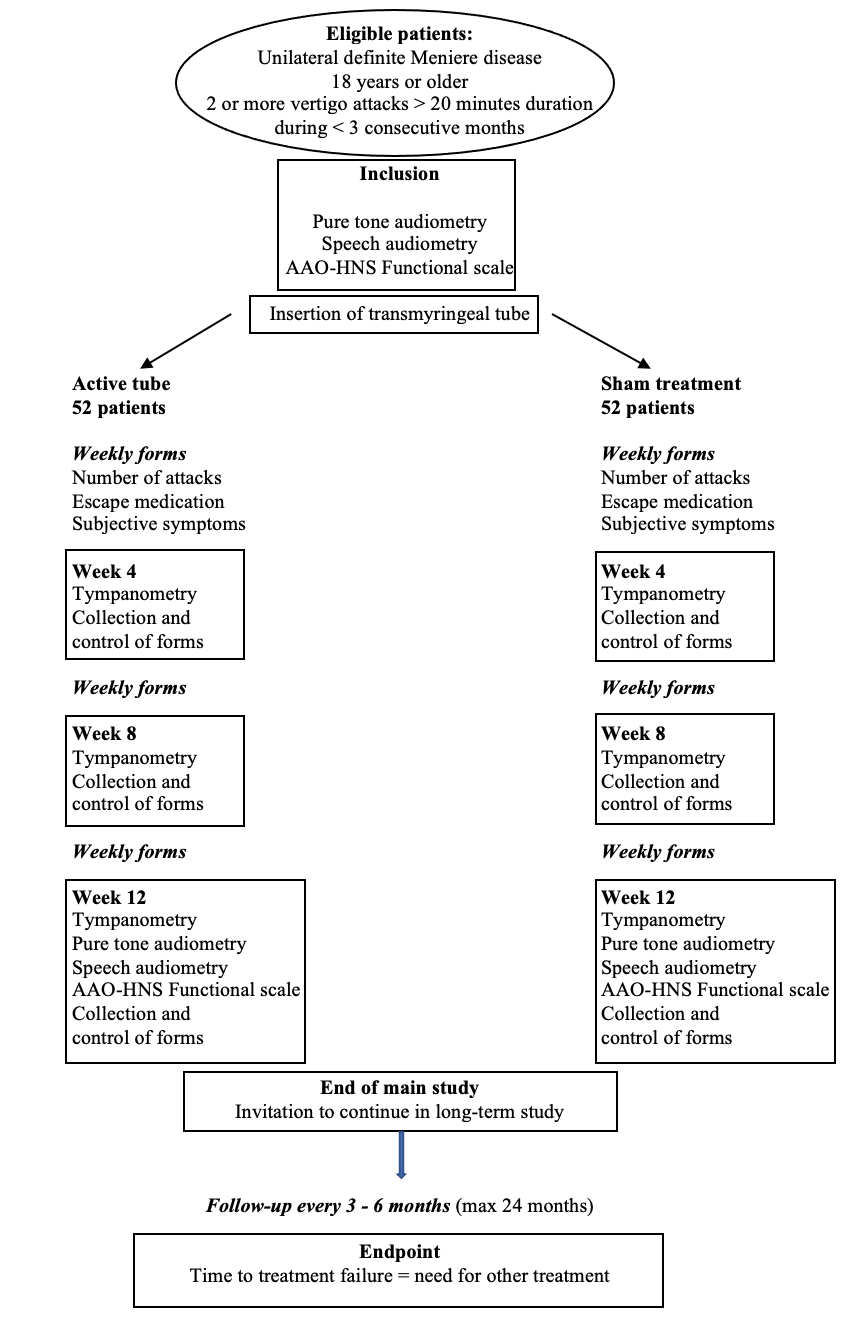


## Flowdiagram for study

|  | Study period | | | | | | | |
| --- | --- | --- | --- | --- | --- | --- | --- | --- |
|  | Enrolment | Allocation | Post-allocation | | | | | Close-out |
| Timepoint | -t_1_ | 0 | t_1_ | t_2_ | t_3_ | t_4_ | t_5_ | t_x_ |
| **Enrolment:** |  |  |  |  |  |  |  |  |
| Eligibility screen | X |  |  |  |  |  |  |  |
| Informed consent | X |  |  |  |  |  |  |  |
| Allocation |  | X |  |  |  |  |  |  |
| **Interventions:** |  |  |  |  |  |  |  |  |
| Ventilation tube insertion |  | X |  |  |  |  |  |  |
| Placebo-treatment |  | X |  |  |  |  |  |  |
| **Assessments:** |  |  |  |  |  |  |  |  |
| Subjective symptom score-scheme* |  | X | X | X | X |  |  |  |
| AAO-HNS functional level scale |  | X |  |  | X | X | X |  |
| Pure-tone and speech audiometry |  | X |  |  | X |  |  |  |

*Patients will be asked to fulfill a weekly subjective symptom score-scheme regarding dizziness, tinnitus, hearing and aural fullness.

**Timeline:**

t_1_: At one month

t_2_: At two months

t_3_: At three months

t_4_: At three to six months from t_3_

t_5_: At three to six months from t_4_

t_6_: At three to six months from t_5_

t_7_: At three to six months from t_6_

t_8_: At three to six months from t_7_

t_9_: At three to six months from t_8_
